# Secondary Contact and Admixture between Independently Invading Populations of the Western Corn Rootworm, *Diabrotica virgifera virgifera* in Europe

**DOI:** 10.1371/journal.pone.0050129

**Published:** 2012-11-26

**Authors:** Gérald Bermond, Marc Ciosi, Eric Lombaert, Aurélie Blin, Marco Boriani, Lorenzo Furlan, Stefan Toepfer, Thomas Guillemaud

**Affiliations:** 1 INRA, UMR 1355 Institute Sophia Agrobiotech, Equipe "Biologie des Populations Introduites", Sophia Antipolis, France; 2 Université de Nice Sophia Antipolis, UMR Institute Sophia Agrobiotech, Equipe "Biologie des Populations Introduites", Sophia Antipolis, France; 3 CNRS, UMR 7254 Institute Sophia Agrobiotech, Equipe "Biologie des Populations Introduites", Sophia Antipolis, France; 4 Institute III, University of Glasgow, Glasgow Biomedical Research Centre, Glasgow, United Kingdom; 5 MBB Unit, International Centre of Insect Physiology and Ecology, Nairobi, Kenya; 6 Servizio fitosanitario regionale, Regione Lombardia, Milano, Italy; 7 Veneto Agricoltura, Legnaro, Italy; 8 CABI Europe_Switzerland, c/o Plant Protection Directorate, Hodmezovasarhely, Hungary; University of Massachusetts, United States of America

## Abstract

The western corn rootworm, *Diabrotica virgifera virgifera* (Coleoptera: Chrysomelidae), is one of the most destructive pests of corn in North America and is currently invading Europe. The two major invasive outbreaks of rootworm in Europe have occurred, in North-West Italy and in Central and South-Eastern Europe. These two outbreaks originated from independent introductions from North America. Secondary contact probably occurred in North Italy between these two outbreaks, in 2008. We used 13 microsatellite markers to conduct a population genetics study, to demonstrate that this geographic contact resulted in a zone of admixture in the Italian region of Veneto. We show that i) genetic variation is greater in the contact zone than in the parental outbreaks; ii) several signs of admixture were detected in some Venetian samples, in a Bayesian analysis of the population structure and in an approximate Bayesian computation analysis of historical scenarios and, finally, iii) allelic frequency clines were observed at microsatellite loci. The contact between the invasive outbreaks in North-West Italy and Central and South-Eastern Europe resulted in a zone of admixture, with particular characteristics. The evolutionary implications of the existence of a zone of admixture in Northern Italy and their possible impact on the invasion success of the western corn rootworm are discussed.

## Introduction

In the early stages of biological invasions, the genetic diversity of populations may be reduced by bottlenecks [1], [2]. This may hinder adaptation to new environmental conditions. However, multiple introductions into the same area followed by admixture may increase genetic variability, offsetting the effect of genetic bottlenecks associated with each introduction [3], [4]. If the various sources are genetically differentiated, this process results in the conversion of interpopulation genetic variation into intrapopulation genetic variation (as reported for the Cuban lizard in Florida, [5]). Conversely, intrapopulation variation may be converted into interpopulation variation if multiple introductions from a single source occur in geographically disconnected areas (e.g., [6]). Secondary contact and admixture between such independently introduced populations can eventually lead to restoration of the genetic variation found in the source population.

Admixture may thus have a positive impact on invasion, through the generation of novel genotypes [3], an increase in intrapopulation genetic variation [5] and heterosis [7]–[9], in which admixed individual fitness exceeds the fitness of the parental populations. Finally, deleterious mutations may be purged from introduced populations during bottlenecks [10], [11]. This may lead to an unusual phenomenon in which multiple introductions followed by admixture may result not only in the restoration of genetic variation, but also in the fitness of the population exceeding that of the source population.

**Figure 1:**
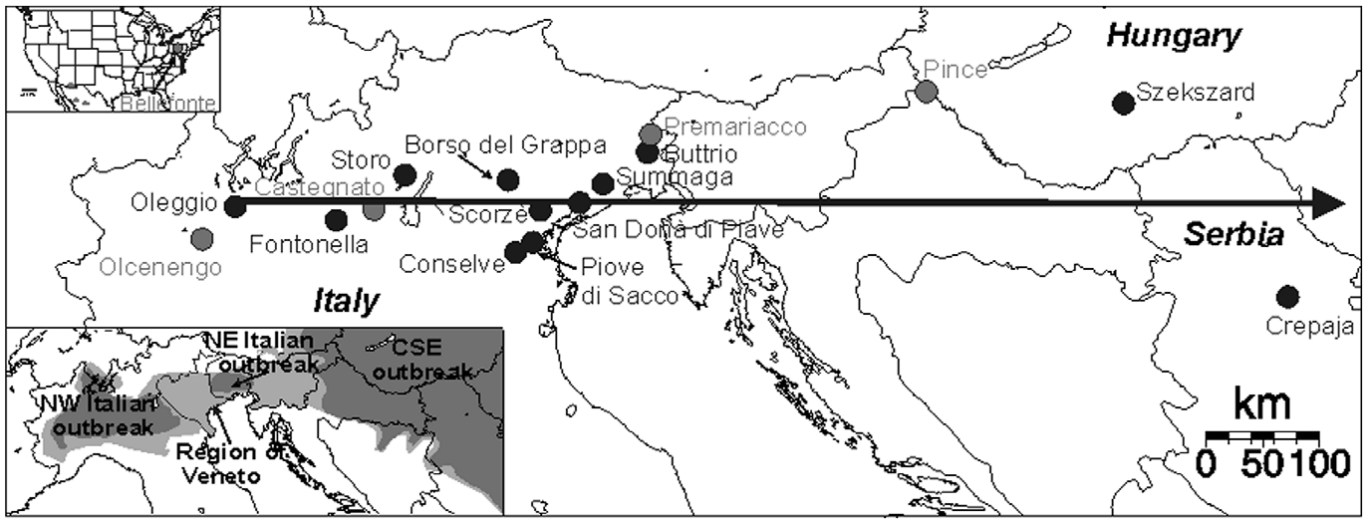
Location of the western corn rootworm (WCR) sample sites in Europe: in North Western (NW) Italy, in the contact zone (Veneto), in North Eastern (NE) Italy, in Central South Eastern (CSE) Europe and in the United States (Pennsylvania). The black circles represent the samples used in the spatial analysis and the black arrow indicates the geographic axis onto which these samples were projected (see Figures 3 and 4). The gray circles represent samples used in the temporal genetic differentiation analysis (collected in 2010 in Northern Italy and CSE Europe) and the sample representative of the native population (collected in 2003 in the United States). In the small map, the distribution areas of WCR before (2005) and after (2010) the secondary contact between the western and eastern outbreak populations are shown in dark gray and light gray, respectively. The dashed delimitation represents the surface of the administrative region of Veneto.

The invasion of Europe by the western corn rootworm (WCR, *Diabrotica virgifera virgifera* (Coleoptera: Chrysomelidae)) has involved multiple introductions into several disconnected geographic areas from a single source population in the northern USA [6], [12]. The WCR is native to Central America [13], [14], probably evolved with corn in Mexico and reached the South Western USA about 3000 years ago, together with its host plant, corn [15]. During the second half of the 20th century, the WCR has rapidly expanded its range across areas of corn production in the American Mid West (see [16] for a review), finally reaching the north-eastern coast of the USA in the late 1980s (see [17] for a review). In the late 20^th^ century and the early 2000s, WCR was introduced into Europe repeatedly from the Northern USA [6], [12]. It first observed near Belgrade Airport in Serbia, in 1992. An international monitoring network has since monitored the distribution and of the WCR and its annual expansion in Europe [18]. There are two types of infested area: i) geographic areas of continuous WCR range expansion in Central and South-Eastern Europe (CSE Europe), North-West Italy (NW Italy) and Bavaria in Southern Germany, corresponding to “invasive” outbreaks and ii) many small disconnected outbreaks that have not persisted over time or expanded geographically, such as area of the North-East Italian (NE Italy) outbreak, which originated from CSE Europe [6], [12], [19]. During the multiple introductions of WCR in Europe from North America [12] strong bottlenecks lead to the formation of several genetically differentiated outbreaks [6]. For example Ciosi et al. [6] found a *F*_ST_ of 0.25 between CSE Europe and NW Italy. The largest outbreak, in CSE Europe, currently covers 16 countries extending from Austria to Ukraine and Southern Poland to Northern Greece. There has also been a westward expansion into eastern parts of Italy. In parallel, the NW Italian invasive outbreak has progressed eastwards. Since 2008, the NW and NE Italian and CSE European outbreak populations have been in close geographic proximity or even in contact, as in the Italian region of Veneto [19]–[21]. The current geographic distribution of WCR is now continuous in Northern Italy [21], [22] (Figure 1). Because the various outbreaks were strongly genetically differentiated, there is probably currently an admixture Northern Italy between the NW Italian, NE Italian and CSE European invasive outbreaks. This situation provides us with an ideal opportunity to follow an evolutionary process in real time *in natura*. The aim of this study was to identify, document and characterize the putative admixture in Northern Italy, following contact between the WCR outbreak populations of NW and NE Italy and that of CSE Europe.

## Materials and Methods

### Sample Collection

No permission is required to collect samples of this species. WCR has no value and is a pest species whose populations are controlled by insecticide treatments or plant rotation wherever they occur. Samples were collected on a west-east transect crossing the outbreak areas of NW Italy, NE Italy and CSE Europe (Figure 1 and Table 1). Adult beetles were caught with sticky traps at the Storo site (NW Italian outbreak) or by hand (net or funnel bound with a muslin bag) at all other sites. The WCR samples used in this study were collected during summer, from June to August, at 12 sites in Italy, Hungary and Serbia (see details in Table 1). At each of the study sites, all the individuals collected were obtained from a single corn field. We distinguished between two types of populations: the parental populations, corresponding to the invasive outbreaks of NW Italy, NE Italy and CSE Europe and the potentially admixed populations in the contact zone between the parental invasive outbreak populations.

**Table 1:** Description of the within-population genetic variation of western corn rootworm samples from Northern Italy and CSE Europe.

Population type	Sample	Outbreak	Origin (Region(Country))	Year of 1^st^ observation	*N*	Sampling date	Distance to Oleggio (km)	*A*		*He*	*F_IS_*	*p-HW*
								*DC*	*AR*			
Parental	Oleggio	NW Italy	Piedmont (Italy)	2000	40	2007	0	3.77 (2.42)	2.70 (1.49)	0.41	0.04	0.40
	Olcenengo	NW Italy	Piedmont (Italy)	2000	30	2010	–	3.39 (2.66)	2.66 (1.60)	0.41	0.10	0.39
	Fontanella	NW Italy	Lombardia (Italy)	2000	33	2007	92	2.77 (1.79)	2.36 (1.32)	0.37	0.06	0.51
	Castegnato	NW Italy	Lombardia (Italy)	2000	30	2010	–	3.00 (1.92)	2.43 (1.32)	0.37	−0.06	0.56
	Storo	NW Italy	Trentino (Italy)	2000	90	2007	151	3.39 (1.85)	2.38 (1.24)	0.39	0.02	0.66
Potentiallyadmixed	Borso del Grappa	–	Veneto (Italy)	2002	20	2009	240	3.15 (1.91)	2.85 (1.64)	0.41	0.04	0.66
	Conselve	–	Veneto (Italy)	2002	20	2009	247	3.00 (1.83)	2.60 (1.48)	0.39	−0.06	0.26
	Piove di Sacco	–	Veneto (Italy)	2002	11	2009	265	3.00 (2.04)	2.85 (1.79)	0.46	0.17	0.39
	Scorzè	–	Veneto (Italy)	2002	10	2009	278	3.15 (1.68)	3.08 (1.56)	0.53	0.00	1
	San Donà di Piave	–	Veneto (Italy)	2002	9	2009	305	3.39 (1.85)	3.37 (1.83)	0.54	0.00	0.33
	Summaga	–	Veneto (Italy)	2002	17	2009	327	3.23 (1.64)	2.94 (1.38)	0.48	0.04	0.46
Parental	Buttrio^X^	NE Italy	Friuli (Italy)	2003	27	2003	–	1.77 (0.73)	1.72 (0.64)	0.29	0.03	0.58
	Premariacco	NE Italy	Friuli (Italy)	2003	29	2010	–	2.70 (1.25)	2.22 (0.81)	0.34	0.08	0.15
	Pince	CSE Europe	Prekmurje (Slovenia)	2003	27	2010		2.77 (1.24)	2.63 (1.15)	0.44	0.08	0.06
	Szekszard	CSE Europe	Tolna (Hungary)	1992	39	2007	780	2.77 (1.24)	2.63 (1.11)	0.46	−0.05	0.98
	Crepaja	CSE Europe	Voïvodine (Serbia)	1992	30	2007	940	2.92 (1.50)	2.69 (1.15)	0.46	0.00	0.59
Native	Bellefonte	Northern USA	Pennsylvania (USA)	1985	42	2003	–	6.10 (4.41)	4.23 (2.16)	0.61	0.05	0.20

**Note:** NW: North-West. NE: North-East. CSE: Central and South-Eastern. Buttrio^X^: this sample was named Friuli in Ciosi et al. [6] and NE Italy in Miller et al. [12]. Distances to Oleggio are provided only for populations included in the spatial analysis. *N*: sample size. *A*: mean number of alleles per locus. *A* was determined by directcounts *(DC)* and allelic richness *(AR)* analysis. *AR* is based on the smallest sample size (N = 8 for several loci of the sample San Donà di Piave). Standard deviations between loci are shown in parentheses. *H*: mean expected heterozygosity [27]. *p-HW*: *p*-values for the exact test of deviation from HW equilibrium.

Additional samples (Figure 1, Table 1) collected in 2010 in the outbreaks of NW Italy, NE Italy and CSE Europe and in 2003 in Northern USA (the native area) were only used for the temporal analysis (see Results section, *Temporal and spatial analyses*). For the Northern USA sample, DNA was extracted from individuals with the BioRad Aqua Pure isolation kit (BioRad, Hercules, CA) according to the manufacturer’s instructions, whereas, for other samples, DNA was extracted with the DNeasy tissue kit (Qiagen, Hilden, Germany), as explained above.

### DNA Extraction and Microsatellite Analyses

All WCR samples were stored in 90–96% ethanol until DNA extraction. Template material for the polymerase chain reaction (PCR) amplification of microsatellite loci was obtained with two different protocols. For the NE Italian sample, the ‘salting out’ protocol of Sunnucks and Hales [23] was used for the rapid extraction of DNA from the head of each individual. For the other samples, we extracted DNA from the thorax or half the body, cut lengthwise, with the DNeasy® tissue kit (Qiagen, Hilden, Germany), according to the manufacturer’s instructions, with an elution volume of 100 µl. Individuals were washed at least three times in 0.065% NaCl before extraction, to remove ethanol from the tissues.

We amplified 13 WCR microsatellite loci (including di and tri-nucleotides [6], [24]) in three separate multiplex PCR performed in a PTC-225 MJ Research thermocycler. The first reaction amplified the DVV-D2, DVV-D4, DVV-D11, and DVV-T2 microsatellites, the second amplified DVV-D5, DVV-D8, DVV-D9 and DVV-ET1, and the third amplified Dba01, Dba05, DVV-T3, DVV-D12 and Dba07.

The thermal cycling conditions were the same for all three reactions and were as described by Miller *et al.* [25]. Forward primers were 5′-labeled with a fluorescent dye for detection of the PCR products on an Applied Biosystems 3130×l Genetic Analyzer. Signal strength was rendered equivalent for different markers, by mixing labeled and unlabeled forward primers in the proportions (labeled:unlabeled) described by Miller *et al*. [25] for the first two sets of markers and in the following proportions for the third set: Dba01 2:1; Dba05 1:1; DVV-T3 1:0; DVV-D12 1:0 and Dba07 1:0. The primers used for DVV-D12 amplified were modified from those originally described by Kim *et al.* [26] (primers used here: F: 5′- GATTCTCAGTAATGGGGAAACG-3′; R: 5′-CACACGCTTTCTCGTAATCTATC-3′This decreased the frequency of null allele detection at this locus to a negligible level. All three multiplex reactions were analyzed as described by Miller *et al.* [25]. All individuals were unambiguously assigned to a diploid multilocus genotype (two peaks per individual at the maximum).

### Classical Statistical Genetics Analysis

Genetic variation within samples was evaluated by determining the mean number of alleles per locus (*A*) and mean expected heterozygosity (*He*) [27]. *A* and *He* were calculated with GENECLASS version 2.0.h [28]. We also calculated *F_IS_* with GENEPOP ver. 4.0.1 [29], [30]. We compared *A* values between population samples by estimating allelic richness (*AR*) based on the smallest sample size, by the rarefaction method [31] implemented in Fstat version 2.9.3 [32]. The various loci are the independent statistical units because they have their own coalescence story. Hence, the differences in *AR* and *He* between samples were assessed in a one-sided Wilcoxon signed rank test (with greater genetic variation in the supposed admixed populations in the contact zone between the parental invasive outbreaks as an alternative hypothesis) with locus as a repetition unit. The significance of the differences between the contact zone (Veneto) and the parental outbreak areas (NW Italy, NE Italy and CSE Europe) was then assessed by combining the probabilities obtained for each sample from Veneto by Fisher’s method [33]. We also used the permutation procedure implemented in Fstat version 2.9.3 to test homogeneity of allelic richness and heterozygosity among samples [32]. We tested for deviation from Hardy-Weinberg equilibrium with the probability test approach implemented in GENEPOP version 4.0.1 [29], [30]. Genetic variation between samples was assessed by calculating Weir and Cockerham’s [34] pairwise *F*_ST_ and by assessing genic differentiation between pairs of samples [35] with GENEPOP version 4.0.1 [29], [30]. When multiple tests were performed to test the same hypothesis, significance levels were lowered according to the Benjamini and Hochberg procedure [36].

### Bayesian Analysis of the Population Structure of WCR

We inferred the genetic structure of the WCR in Northern Italy, using the Bayesian method implemented in STRUCTURE version 2.3.3 [37]. We performed 20 runs for each value of the number of clusters (*K*), defined as lying between 1 and the total number of sampling sites. Each run consisted of a burn-in of 5×10^4^ iterations, followed by 10^5^ iterations. We used the admixture model of ancestry together with the correlated allele frequencies model [38], with and without the use of sampling location as prior information [39]. Default values were maintained for all other parameters. *K* was estimated with Evanno’s Δ*K* statistic [40], which is based on the rate of change in log-likelihood between successive values of *K* and the variability of log-likelihood between different runs. The most likely run was then represented with DISTRUCT version 1.0 ([41], see Figure 3).

Genetic differentiation was found between two samples collected from NE Italy in 2003 and 2010 (see details in Results, section *Temporal and spatial analyses* and Figure 1). We therefore removed the Buttrio (NE Italy) site from the Bayesian analysis and analyses of geographic population structure. However, as the NE Italian outbreak population may be a source of the Veneto populations, we considered the NE Italian sample from 2003 in the Approximate Bayesian Computation (ABC) analysis.

### Geographic Analyses and Estimation of Admixture Rate

We tested the effect of geographic distance to Oleggio (the sampling site at the extreme west of the studied area, defined here as the geographic reference point) on microsatellite allelic frequencies. We defined a geographic axis passing through the various sampling sites and we then projected each sampled population orthogonally on this axis (Figure 1). The projected coordinates were then used to calculate the distance between each sample and the Oleggio sample. For each locus, the effect of this distance on allelic frequencies was assessed with a generalized linear model in SAS version 9.1.3 [42]. A multinomial distribution was chosen for the residual error and a cumulative logit function was used as the link. In cases of overdispersion an *F* test was carried out rather than a χ^2^ test to evaluate the effect of distance [43].

The admixture rate was estimated (i) as described by Choisy *et al.* [44], with Oleggio and Crepaja as representative samples of the parental populations (these sites are the closest to the sites at which WCR was first observed in each parental outbreak), or (ii) with the coefficient of coancestry *Q,* calculated with STRUCTURE version 2.3.3 [37], [38].

### ABC Analysis of Historical Scenarios of WCR Invasion in Northern Italy

For each sampling site within the contact zone (i.e. for each of the six Venetian target samples), we conducted six independent ABC analyses comparing various historical scenarios (Figure S1, Supporting information) differing in terms of the source of the population at the Venetian site concerned. Each parental outbreak population (CSE Europe, NE and NW Italy) was represented by the sample obtained from the site closest to the location at which the first observation for the outbreak concerned was reported (Crepaja, Buttrio and Oleggio for the CSE Europe, NE and NW Italy outbreaks, respectively). We thus considered a total of nine scenarios for each Veneto sites, according to the origin of the population: (i) NW Italy outbreak (represented by Oleggio), (ii) NE Italy outbreak (represented by Buttrio), (iii) CSE Europe outbreak (represented by Crepaja), (iv, v, vi) all three possible scenarios of a single admixture between NW Italy, NE Italy and CSE outbreak populations (vii, viii and ix) all three possible scenarios of a double admixture between NW Italy, NE Italy and CSE Europe outbreak populations. The history of the putative source populations was defined in accordance with published reports [6], [12]. The NW Italy and CSE Europe populations were considered to have originated independently from North America, whereas the NE Italy population was considered to be derived from the CSE Europe population.

ABC analyses [45] were performed with DIYABC version 1.0.4.40 [46], with parameter values drawn from the prior distributions described hereafter and obtained by simulating 10^6^ microsatellite datasets for each competing scenario.

Simulated and observed datasets were summarized with summary statistics, which were then used to calculate Euclidean distances between the simulated and observed datasets. We then estimated the posterior probabilities of the competing scenarios by polychotomous logistic regression [47] on the 1% of the simulated datasets closest to the observed dataset, after reducing the parameter space by a linear discriminant analysis (LDA) approach [48]. In cases of an overlap between the confidence intervals of the two largest posterior probabilities (each corresponding to a particular historical scenario), we repeated the ABC analyses with the two competing scenarios only.

We used the summary statistics describing genetic variation within and between populations generally used for approximate Bayesian computation analyses [47], [49]–[51]. For each population and each population pair, we used the mean number of alleles per locus, mean heterozygosity, the mean ratio of the number of alleles to the range of allelic size, the *F*_ST_ between pairs of populations and mean individual assignment log-likelihoods of individuals from population *i* being assigned to population *j* and the maximum likelihood estimates for admixture proportions.

The prior distributions of the historical, demographic and mutational parameters used in the ABC analysis were as follows: *N_si_*, the effective population size of the WCR American (USA) or European (CSE Europe, NW Italy, NE Italy and Veneto) source populations and *N_sG_,* the effective size of the ghost population (an unsampled population called the “ghost population” was included in the analysis for “double admixture” scenarios between the NE and NW Italy and CSE outbreaks; this “ghost population” is the result of a single admixture between two of the three putative source populations, depending on the scenario), were drawn from a uniform distribution bounded by 1000 and 20000 (Uniform[1000; 20000]); *N_Fi_* and *N_FG_,* the effective number of founders of the European populations, were drawn from a Uniform[1; 100] distribution; the bottleneck duration of population *i*, *BD_i_*, was drawn from a Uniform[1]; [5] population. Each introduced population *i* (*i*  = 1, 2, 3, 4 for Veneto (the contact zone in Northern Italy), NE Italy, NW Italy and CSE Europe, respectively) was founded by individuals originating from its source population *t_i_* (and *t_G_* for the ghost population) generations before the present (i.e. 2010, year of the study). As WCR is univoltine, *t_i_* and *t_G_* are also the number of years before the present. *t_1_* and *t_G_*, *t_2_*, *t_3_*, and *t_4_* were drawn from between one and five generations (or years) before the date of the first observation (see Table 1) and were thus drawn from Uniform [9]; [13], [8]; [12], [11]; [15] and [19]; [23] distributions, respectively. In all double admixture scenarios, we fixed *t_1_*<*t_G_*. We used a generalized stepwise mutation model (GSM [52]). A mean mutation rate across loci, *μ,* was first drawn from a Uniform [10^−4^; 5×10^−3^] distribution, and single locus mutation rates, *µ_k_,* were then drawn from gamma distributions with a mean of *μ* and a shape parameter of 2 (rate = 2/μ). For each locus, the coefficient *P* of the geometric distribution of repeat units by which a new mutant allele differs from its ancestor was drawn from an exponential distribution with a mean of 0.22.

We evaluated the ability of ABC to select the true scenario correctly, by analyzing test datasets simulated from known competing scenarios. For each scenario, one hundred such datasets were simulated with parameter values drawn from the same probability distributions as the priors. The posterior probabilities of each competing scenario were estimated for each simulated test dataset, with the same ABC procedure as described above, and were used to calculate type I and II errors in the selection of scenarios. Type I error is the proportion of simulations in which the scenario considered is excluded but is actually the true one. Type II error is the proportion of simulations in which the scenario considered is selected but is not the true one. Small Type 2 errors provide good confidence in the results even if the Type 1 errors are large.

## Results

### Temporal and Spatial Analyses

The main body of the present study concerns spatial analyses of WCR genetic variation. When the samples were not collected within the same year as in our case, a procedure must guarantee that no temporal confounding effect occurs. Such a temporal effect was hence tested using samples collected within the same area at various dates (Lombardy, Piedmont, Friuli and CSE Europe). Locations showing a significant temporal effect were removed from the spatial analysis.

No temporal differentiation was found between samples collected in the NW Italy outbreak in Piedmont (Oleggio, 2007, and Olcenengo, 2010), in Lombardia (Fontanella, 2007, and Castegnato, 2010) and in the CSE Europe outbreak (Hungary (Tolna) and Serbia (Voïvodine) in 2007 and Slovenia (Prekmurje) 2010; *p*>0.08 for all pairwise comparisons considered). These locations were thus kept and the 2007 samples were arbitrarily chosen for subsequent spatial analyses. A significant temporal differentiation was found in Friuli (Buttrio, 2003, and Premariaco, 2010; *p = *0.001). Friuli was thus not considered for the spatial analyses.

The Friuli sample of Buttrio was sampled in 2003, i.e. before the contact between the NW, NE Italian and CSE European outbreaks in 2008. Hence, it properly describes the north eastern Italian outbreak before any contact between the various outbreaks. Buttrio was therefore used in the ABC historical scenario comparison.

### Genetic Variation within and between Populations

**Figure 2:**
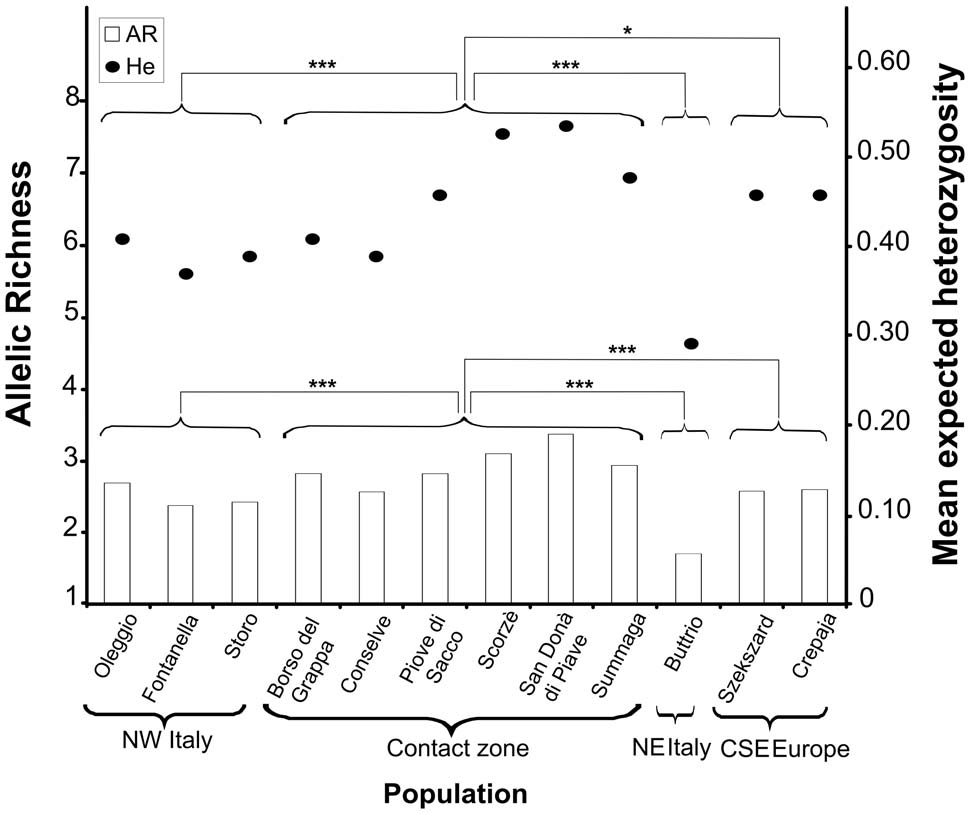
Comparison of genetic variation between potentially admixed western corn rootworm samples (Veneto) and their putative parental (NE and NW Italy and CSE Europe) populations. Gray diamonds indicate the mean expected heterozygosity *(He)* for the various samples on the right axis, whereas white bars indicate allelic richness (*AR*) on the left axis. Significant differences in mean expected heterozygosity and allelic richness between samples are indicated by asterisks (based Fisher’s method for combining probabilities [33]).

Overall, the European WCR populations displayed moderate polymorphism, with 5.31 (SD = 3.71) alleles per locus over all samples. Within samples, the mean number of alleles was between 1.77 (SD = 0.73) for Buttrio in NE Italy and 3.77 (SD = 2.43) in Oleggio in NW Italy (Table 1). Mean expected heterozygosity (*He*) was low to moderate and varied from 0.29 for Buttrio in NE Italy to 0.54 for San Donà di Piave in Veneto (Table 1). *F_IS_* estimates were low and no significant deviation from Hardy-Weinberg equilibrium was observed (Table 1). Samples from Veneto contained a significantly larger number of alleles than parental samples (Fisher’s method for the combination of probabilities, χ^2^ = 121.28; df = 36; p<10^−5^; χ^2^ = 66.31; df = 12; p<10^−5^ and χ^2^ = 61.24; df = 24; p<10^−5^ for the comparisons “Veneto/NW Italy, Veneto/NE Italy and Veneto/CSE Europe”, respectively; Figure 2) and heterozygosity was significantly greater for samples from Veneto than for all other samples (Fisher’s method for the combination of probabilities, χ^2^ = 110.97; df = 36; p<10^−5^; χ^2^ = 60.22; df = 12; p<10^−5^ and χ^2^ = 40.81; df = 24; p<0.05, for the comparisons “Veneto/NW Italy, Veneto/NE Italy and Veneto/CSE Europe”, respectively; Figure 2). Permutation tests performed with Fstat revealed a significantly larger allelic richness (one sided-test, p = 4×10^−3^) and a marginally significantly larger heterozygosity (one sided-test, p = 0.062) in Veneto compared to parental samples. Most pairwise comparisons of samples (92%) showed significant genetic differentiation (Table 2). Veneto samples generally displayed significant genetic differentiation from i) the samples from the three putative parental outbreak populations and ii) from each other (Table S1).

**Table 2:** Pairwise *F*_ST_ estimates [34] between Northern Italian and Central and South-Eastern European samples of the western corn rootworm.

Population	North-West Italy			Contact zone						North-East Italy	Central and South-Eastern Europe
Sample site	Oleggio	Fontanella	Storo	Borso del Grappa	Conselve	Piove di Sacco	Scorzè	San Donà di Piave	Summaga	Buttrio	Szekszard
Fontanella	0.00										
Storo	**0.01**	0.01									
Borso del Grappa	**0.03**	**0.02**	**0.02**								
Conselve	**0.03**	0.02	**0.01**	**0.04**							
Piove di Sacco	**0.02**	**0.02**	**0.01**	0.01	**0.03**						
Scorzè	**0.11**	**0.12**	**0.12**	**0.06**	**0.11**	**0.06**					
San Donà diPiave	**0.08**	**0.10**	**0.10**	**0.06**	**0.09**	**0.06**	**0.03**				
Summaga	**0.24**	**0.27**	**0.28**	**0.20**	**0.29**	**0.20**	**0.08**	**0.08**			
Buttrio	**0.38**	**0.42**	**0.40**	**0.36**	**0.46**	**0.38**	**0.29**	**0.27**	**0.14**		
Szekszard	**0.25**	**0.27**	**0.28**	**0.19**	**0.29**	**0.20**	**0.08**	**0.11**	**0.02**	**0.12**	
Crepaja	**0.25**	**0.26**	**0.27**	**0.17**	**0.28**	**0.19**	**0.07**	**0.10**	**0.02**	**0.15**	0.00

**Note:** Significant pairwise differentiation tests after correcting for the false-positive rate by the procedure of Benjamini and Hochberg [36] are shown in bold typeface. From left to right, samples are ordered from west to east, from Italy to Serbia.

### Bayesian Analysis of the Structure of WCR Samples in Italy and CSE Europe

**Figure 3:**
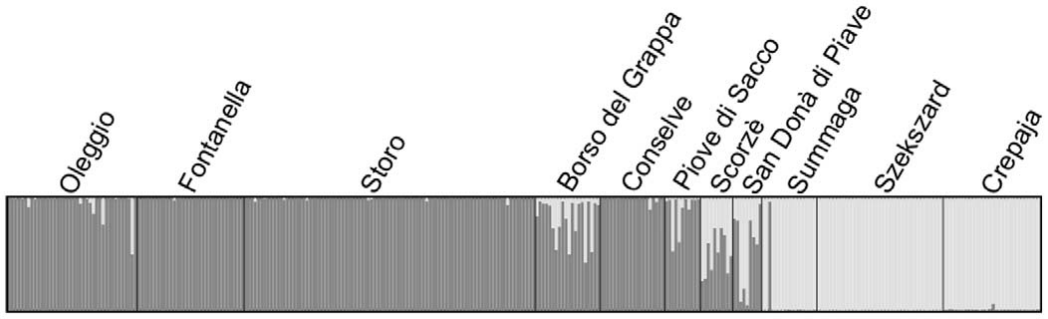
Population structure, based on 13 microsatellite loci from WCR, as estimated by genotypic clustering in STRUCTURE. Assignment of 319 individuals to *K* = 2 genetically distinct clusters. Individuals are represented by a vertical bar, grouped by sampling location (the name of which is above the plot). The proportion of dark gray in each bar indicates the individual coefficient of coancestry (or admixture rate).

The number of genetic clusters *(K)* was estimated at *K* = 2, whatever the model used. The analysis of coancestry coefficients (*Q*) (for the model with the use of sampling location as prior information) (Figure 3) indicated that (i) the vast majority of individuals from the west of the transect belonged to a single cluster (with *Q* >0.86 for 161 of 163 individuals) (ii) individuals from the east belonged to another cluster (with *Q <*0.07 for all individuals) and that (iii) a large number of individuals from Scorzè, San Donà di Piave, Borso del Grappa and Piove di Sacco are admixed between both clusters (with *Q* between 0.20 and 0.80). One individual from Summaga, a population belonging to the eastern genetic cluster, was assigned to the western genetic cluster (*Q* = 0.97).

### Detection of Clines of Allelic Frequencies among WCR Samples in Italy and CSE Europe

**Figure 4:**
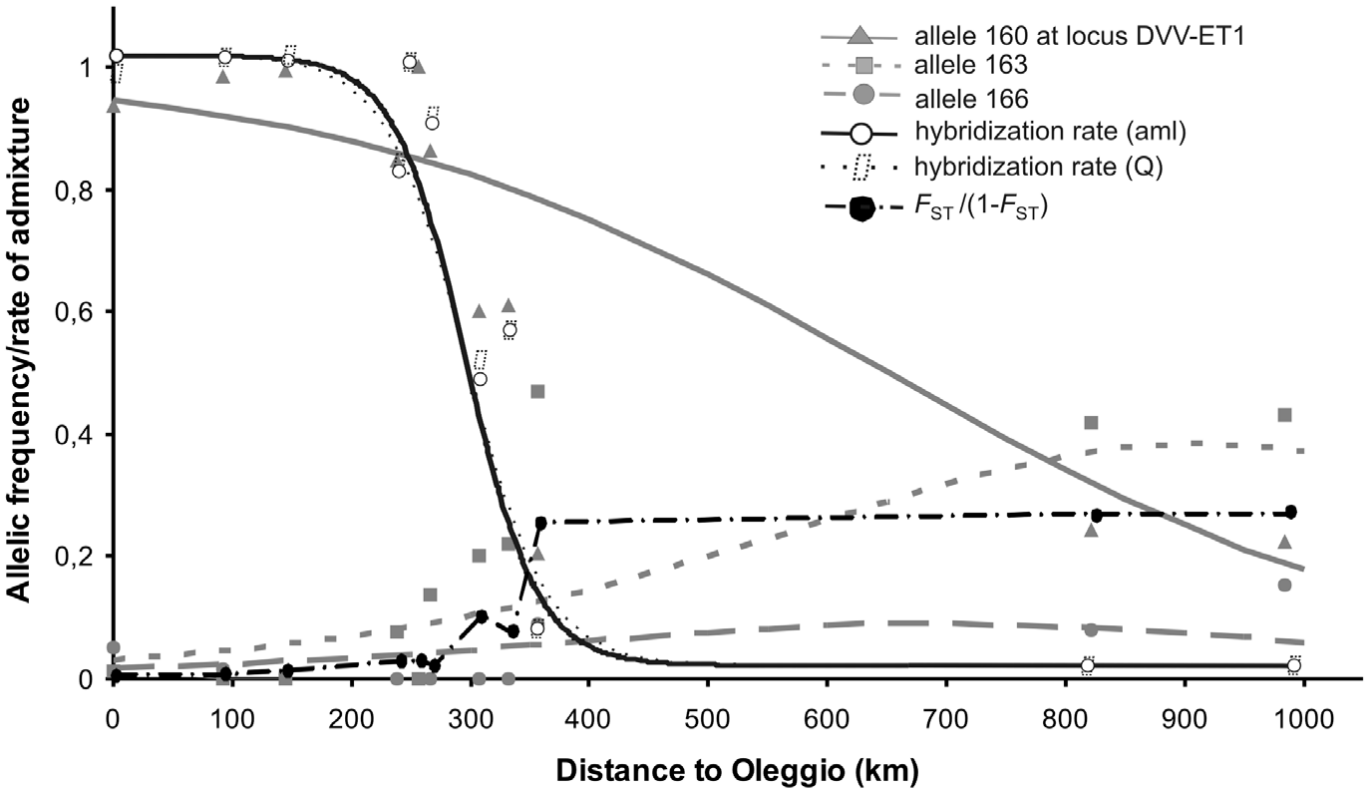
Observed and fitted values of allelic frequencies at microsatellite locus DVV-ET1, of rates of admixture and index of genetic differentiation (*F*_ST_/(1-*F*_ST_)) relative to Oleggio, as a function of geographic distance to the Oleggio site in North-West Italy. Observed values are indicated by symbols whereas fitted values are indicated by lines. The fit for each allele corresponds to the generalized linear model described in the materials and methods. Three of the four alleles present at this locus are represented because the frequency of the 4^th^ allele is simply the complement of those of the three others. Similar pattern were observed for 5 other loci. A sigmoid function was fitted to the admixture rates. For (Fst/(1-Fst)), only observed values are shown.

A significant effect of geographic distance on allelic frequencies was found for six of the 13 loci studied (*p*<0.0033 for each of these 6 loci; see Figure 4 for example). Distance had a significant effect over all loci (Fisher’s method for the combination of probabilities, χ^2^ = 113.62, df = 26, *p*<10^−5^). The admixture rates obtained by the two different methods (the method of Choisy *et al.* [44] and the coancestry coefficient from STRUCTURE) were very similar and closely matched a sigmoid logit model (*R^2^* = 0.90 for both methods). Figure 4 shows that the center of the cline is located about 310 km from Oleggio, in Veneto, and that this cline is about 100 km wide.

The effect of the geographic distance on the *F*_ST_/(1-*F*_ST_) between each sample and Oleggio, the sample located in the extreme west of the studied area was significant (Spearman rank-order correlation test, p<10^−5^, Figure 4).Genetic differentiations compared to Oleggio sample are very low for western samples (NW Italy outbreak) and substantial for eastern samples, with a *F*_ST_ of about 0.25 in CSE Europe outbreak. A sharp discontinuity of *F*_ST_/(1-*F*_ST_) is observed in the contact zone, i.e. in Veneto, where values are intermediate.

### ABC Analysis of Historical Scenarios of WCR Invasion in Northern Italy

The ABC procedure was used to calculate the posterior probabilities of the evolutionary scenarios describing the origin of each population sampled in Veneto. According to the highest probabilities with non overlapping confidence intervals, four of the six Veneto samples probably originate from admixture events: samples from Scorzè and San Dona di Piave probably originate from admixture between the outbreak populations of NW Italy and CSE Europe (Table 3); samples from Borso del Grappa and Summaga probably result from double admixture between NW Italy, NE Italy and CSE Europe outbreak populations and samples from Conselve and Piove di Sacco have a simple origin in NW Italy.

**Table 3:** ABC analysis of the nine historical scenarios describing the history of each population sampled in the secondary contact zone (Veneto).

	Historical scenarios										
	Without admixture			Single admixture			Double admixture			Errors	
Target sample in the secondary contact zone	NW Italy	NE Italy	CSE Europe	NW Italy/NEItaly	NE Italy/CSE Europe	NW Italy/CSE Europe	(NW Italy/NE Italy)/CSE Europe	(CSEEurope/NEItaly)/NWItaly	(CSE Europe/NW Italy)/NE Italy	Type I	Type II (min–max)
Borso del Grappa	0.19 [0.18;0.21]	0.0 [0.0;0.0]	0.0 [0.0;0.0]	0.17 [0.16;0.18]	0.0 [0.0;0.0]	0.31 [0.30;0.33] (0.37)[0.36;0.38]	0.02 [0.02;0.02]	0.29 [0.28;0.31] **(0.63) [0.62;0.64]**	0.01 [0.01;0.01]	0.66	0.08 (0.0–0.23)
Conselve	**0.99** **[0.98;0.99]**	0.0 [0.0;0.0]	0.0 [0.0;0.0]	0.0 [0.0;0.0]	0.0 [0.0;0.0]	0.0 [0.0;0.0]	0.0 [0.0;0.0]	0.01 [0.0;0.01]	0.0 [0.0;0.0]	0.10	0.04 (0.0–0.12)
Piove di Sacco	0.27 [0.21;0.32] **(0.56) [0.55;0.58]**	0.0 [0.0;0.0]	0.0 [0.0;0.0]	0.18 [0.14;0.21]	0.0 [0.0;0.0]	0.22 [0.18;0.26]	0.0 [0.0;0.0]	0.33 [0.28;0.37] (0.44) [0.42;0.45]	0.0 [0.0;0.0]	0.06	0.05 (0.0–0.14)
Scorzè	0.0 [0.0;0.0]	0.0 [0.0;0.0]	0.0 [0.0;0.0]	0.07 [0.06;0.09]	0.0 [0.0;0.0]	**0.54** **[0.49;0.59]**	0.10 [0.08;0.12]	0.26 [0.22;0.30]	0.03 [0.02;0.03]	0.58	0.09 (0.0–0.23)
San Donà di Piave	0.01 [0.0;0.01]	0.0 [0.0;0.0]	0.0 [0.0;0.0]	0.17 [0.14;0.20]	0.0 [0.0;0.0]	**0.41** **[0.37;0.46]**	0.07 [0.06;0.09]	0.31 [0.26;0.34]	0.03 [0.02;0.04]	0.43	0.07 (0.02–0.30)
Summaga	0.0 [0.0;0.0]	0.0 [0.0;0.0]	0.03 [0.02;0.04]	0.02 [0.01;0.02]	0.01 [0.0;0.01]	0.13 [0.10;0.15]	**0.61** **[0.57;0.66]**	0.11 [0.09;0.14]	0.09 [0.07;0.11]	0.67	0.06 (0.01–0.15)

Posterior probabilities are given, with their confidence intervals and associated type I and type II errors.

**Note:** 95% confidence intervals are indicated in brackets. Values in parentheses are the new posterior probabilities of scenarios re-analyzed in ABC due to the overlap between confidence intervals. The highest probability values are shown in bold typeface and indicate the best scenario. NW: North-West. NE: North-East.

CSE: Central and South-Eastern.

Some of the largest posterior probabilities were only moderate (Table 3) and some type I errors were large (four of the six values are between 0.43 and 0.67). However, as explained in the material and method, low type 2 errors (<0.09, Table 3) suggest that we can have a high degree of confidence in the choice of scenario.

## Discussion

Since the first observation of the CSE European outbreak of WCR near Belgrade in 1992, a monitoring network has followed the progression of this pest in Europe [17], [18], [22]. No contact between the NW Italian, the NE Italian and CSE European outbreak populations was detected until 2008, since when the distribution of WCR in Northern Italy has been continuous, extending from NW Italy to CSE Europe [20], [21]. Population genetic analysis of the WCR collected in the zone of secondary contact between the NW Italian and CSE outbreak populations revealed a high degree of genetic heterogeneity, determined principally by geography. Our results confirm show the existence of a zone of admixture in Northern Italy, with the occurrence of admixture between highly differentiated populations.

### A Contact and Zone of Admixture in Northern Italy, in the Veneto Region

The current contact zone between the different European outbreak populations (NW Italy, NE Italy and CSE Europe) is located in Veneto. This zone has been intensively monitored with pheromone traps since the first captures of WCR in the Venetian region in 1998, leading to the implementation of an eradication program. Since 2008, the geographic distribution of WCR has been continuous in Northern Italy [21], [22] due to contact between the outbreak populations of NW and NE Italy and CSE Europe [19]–[21].

Our analysis of the population genetic structure of WCR in Northern Italy and CSE Europe showed that the WCR populations sampled in Veneto resulted mostly from contact and admixture between the NW Italian and CSE European outbreak populations. The admixture analysis, the Bayesian analysis of population genetic structure and the ABC analysis of scenario choice all indicate that the Veneto region is a zone of admixture, containing admixed individuals. The genomes of individuals sampled in Veneto could be attributed to both the genetically different clusters of NW Italy and CSE Europe, with various levels of admixture. Moreover, the rate of admixture between the NW Italian and CSE European outbreaks varied evenly across a gradient from west to east.

According to the ABC analysis, most samples from the western part of Veneto have a simple NW Italian origin, whereas all the other sample populations result from single or double admixture events between the three parental outbreak populations (NE and NW Italy and CSE Europe). Double admixture between individuals originating from distant populations (here, the three parental populations) accounted for the origin of the most westerly sample from Veneto (Borso del Grappa). This implies that individuals from the eastern outbreak populations (the NE Italy and CSE Europe outbreaks) migrated to the western part of Veneto, suggesting that long-distance dispersal occurs. Long-distance dispersal is also suggested by the detection of an individual genetically assigned to the western genetic cluster in Summaga, a population from the eastern genetic cluster. Recent studies have suggested that long-distance dispersal is common in WCR and that invasive populations of WCR are expanding through stratified dispersal [17], [53].

The occurrence of a cline in the Veneto region in 2009, even with such a large width (about 100 km), is consistent with the short period of time between secondary contact (2008) and sampling (2009). The WCR has a considerable capacity for dispersal, as shown by the rapid rate of expansion of the CSE Europe outbreak population (60–100 km (60–100 km per year [54])). A long period between sampling and contact would therefore probably have led to the homogenization of microsatellite frequencies over space [55].

A thorough clinal analysis of WCR in Veneto is required, to estimate dispersal parameters (e.g., [56]). Under the effect of dispersal alone, allelic frequencies tend to become homogeneous over the zone of admixture, so the slope of the cline depends on the strength of dispersal and contact time (e.g., [55]). The speed at which the slope decreases over time is thus a direct function of the dispersal intensity [57]. Temporal analysis of the North Italian zone of admixture should therefore provide an estimate of the dispersal capacities of WCR, as recently reported for *Biston Betularia* by Saccheri *et al.* [58].

It is noteworthy that biological invasions may provide numerous opportunities to estimate dispersal parameters using such analysis of frequency cline. It is now admitted that multiple invasions are frequent [59], [60]. This can lead to situations, as in WCR, in which recently introduced populations display large neutral genetic differentiation and will eventually merge during their geographical expansion. This evolutionary scenario, that allows estimation of dispersal through clinal analysis, probably occurred in the case of the green crab *Carcinus maenas* in the north Eastern American coasts [60], [61]. It also probably occurred in the plant pathogenic fungus *Mycosphaerella fijiensis* in Africa [62], and is currently occurring in the Asian ladybeetle *Harmonia axyridis* in North America [51], [63]. Even though biological situations allowing such dispersal estimation are abundant, the literature provides no example of such studies to our knowledge. As discussed by Ciosi et al. [6] this lack may result from a simple difficulty: The rapid spatial spreading, the late observation and the late sampling of the invasive populations likely result in the observation of a single genetically homogenized population, with no observable frequency clines.

In cases of selection acting against hybrids, the clines may remain stable over time. The sampling of WCR in Northern Italy in consecutive years, followed by a temporal analysis of cline shape (slope and width), may thus provide information about the balance between dispersal and selection [64]–[66] in this pest species.

### Evolutionary Implications of the Existence of a WCR Admixture Zone in Northern Italy

We found that genetic variation was greater in the zone of admixture than in the parental outbreak areas. Moreover, the genetic variation within the zone of admixture is approaching that of the Northern USA source population and thus displays substantial restoration of the genetic variation lost during the introduction and establishment of the invasive outbreaks. Indeed, the mean allelic richness and the mean heterozygosity of the zone of admixture represent 70% and 77% of that of the Northern USA source population, respectively, whereas the NW Italian, the NE Italian and CSE Europe outbreaks display a mean of 59, 41 and 63%, of the allelic richness and 64, 47 and 76% of the heterozygosity found in the Northern USA, respectively (see more details in the Figure S2). This study and previous population genetics studies of European populations of WCR provide information about the way in which this increase occurred, because it has been possible to observe (i) a loss of genetic variation during the founding of most of independently introduced populations [6], [12], and (ii) admixture between the outbreak populations leading to a partial restoration of genetic variation (this study). An increase in neutral genetic variation was observed, but our results provide no information about differences in the phenotypic variability and fitness of WCR from the zone of admixture and from the parental areas. Recent invasion studies have reported example of a positive effect of admixture on the invasive capacity of animals such as the Asian ladybird *Harmonia axyridis* [67] and the freshwater snail *Melanoides tuberculata* [8], and of plants [3], such as *Silene vulgaris* [68]. In WCR, further quantitative genetics studies of life history traits (such as fertility, longevity and dispersal) are required to determine whether admixture has been and is currently an advantage for the invasion of Europe by this species.

## Supporting Information

Figure S1 **Graphical representation of the competing scenarios used for the ABC analyses on our European dataset.** Scenarios (i), (ii) and (iii) represent the “simple origin” scenarios, scenarios (iv), (v) and (vi) are “single admixture” scenarios and (vii), (viii) and (ix) are “double admixture” scenarios. Historical and demographic parameters were identical for all introduction models. Time 0 is the present and represents the year of the study (2010). The Veneto population was founded *t_1_* generations before the present, had an effective number of founders *NF_Veneto_* with the population remaining at this size for *BD_1_* generations (bottleneck duration) and then reached a larger stable effective population size *NS_Veneto_*. The putative source populations, the Central South-Eastern European (CSE Europe) and North-West (NW) Italian outbreak populations, diverged from the USA population *t_4_* and *t_3_* generations ago with an effective numbers of founders *NF_CSE_* and *NF_NWItaly_*, bottleneck durations *BD_4_* and *BD_3_* and stable effective population sizes *NS_CSE_* and *NS_NW Italy_*, respectively. The North-East (NE) Italian population was founded *t_2_* generations ago from the CSE European population, with an effective number of founders *NF_NEItaly_*, a bottleneck duration *BD_2_* and an effective population size *NS_NEItaly_*. When admixture occurs, the admixture rates *ar* and 1-*ar* are the genetic contribution of each of the source populations to the origin of the Veneto population. An unsampled population called the “Ghost population” was included into the analysis to allow “double admixture” scenarios (scenario (vii), (viii) and (ix)) between NE and NW Italy and CSE outbreak populations. This “Ghost population” is the result of a single admixture between two of the three putative source populations (the populations involved depend on the scenario), *t_G_* generations ago (*t_G_*>*t_1_* and *t_G_*<*t_2_*), with an effective number of founders *NF_GhostPopulation_*, a bottleneck duration *BD_G_* and an effective population size *NS_GhostPopulation_*. The rates of admixture corresponding to the “Ghost population” are *ar_G_* and 1-*ar_G_*. For all models, populations were assumed to be isolated from each other, with no exchange of migrants.
(TIF)

Figure S2 **Percentage of the mean expected heterozygosity (** ***He*** **) and allelic richness (** ***AR*** **) of the Northern USA source population (Bellefonte, Pennsylvania) for each sample from the three putative parental outbreak areas (NW and NE Italy and CSE Europe) and for samples from Veneto.** Dark gray bars indicate the mean expected heterozygosity (*He*) of the various samples, whereas light gray bars correspond to their allelic richness (*AR*).
(TIF)

Table S1 **Mean pairwise** ***F_ST_*** **comparisons between Northern Italian (NW Italy, Veneto and NE Italy) and Central and South-Eastern European (CSE Europe) samples of western corn rootworm (WCR).**
(DOC)
