# Supplementary figures and images for: Secondary Contact and Admixture between Independently Invading Populations of the Western Corn Rootworm, Diabrotica virgifera virgifera in Europe
Source: PLoS One. 2012 Nov 26;7(11):e50129. doi: 10.1371/journal.pone.0050129 (PMC3506547; doi:10.1371/journal.pone.0050129)

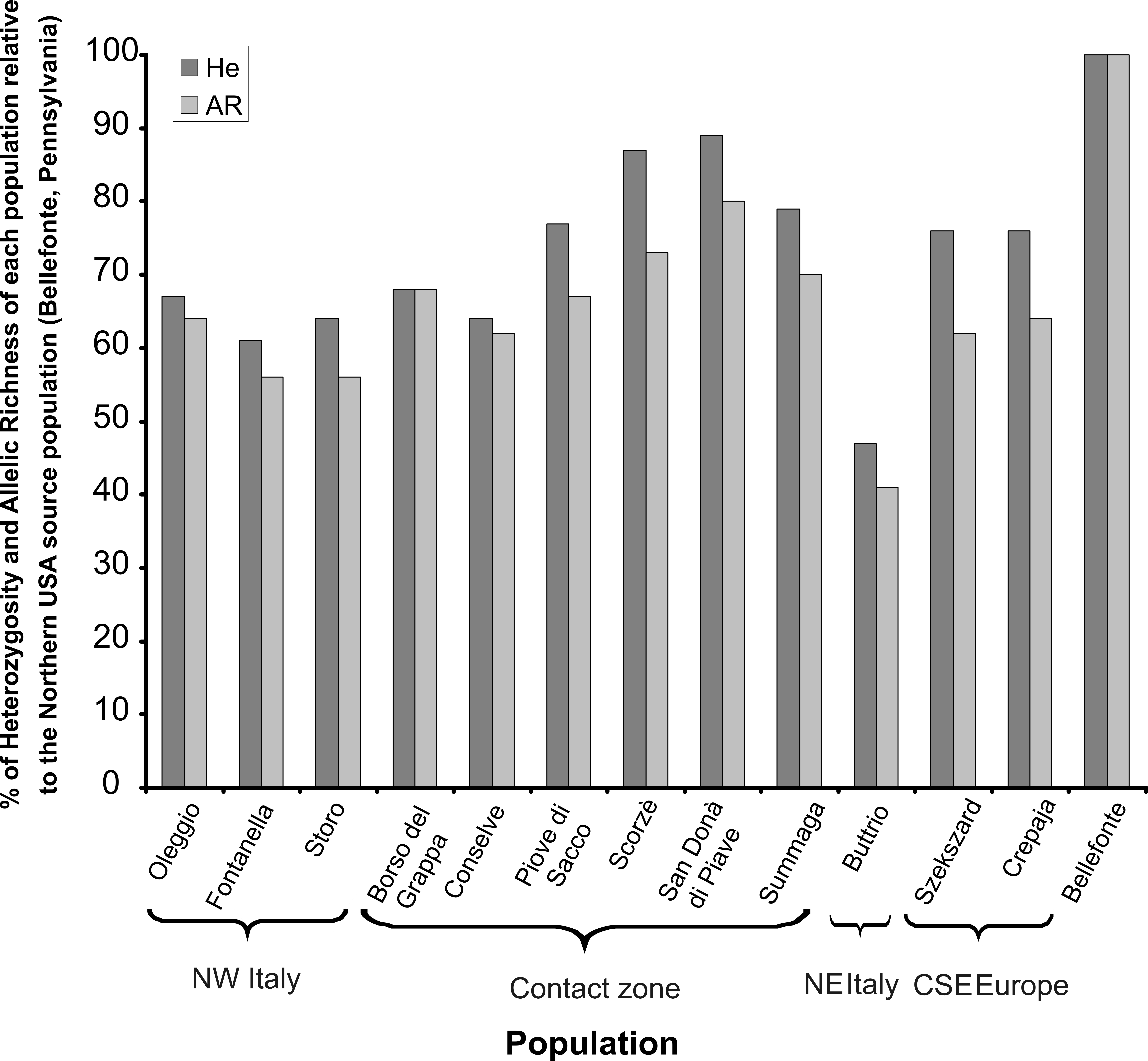

Supplement: Figure S2 — Percentage of the mean expected heterozygosity ( He ) and allelic richness ( AR ) of the Northern USA source population (Bellefonte, Pennsylvania) for each sample from the three putative parental outbreak areas (NW and NE Italy and CSE Europe) and for samples from Veneto. Dark gray bars indicate the mean expected heterozygosity (He) of the various samples, whereas light gray bars correspond to their allelic richness (AR). (TIF) [file pone.0050129.s002.tif]
