# Supplementary material for: Secondary Contact and Admixture between Independently Invading Populations of the Western Corn Rootworm, Diabrotica virgifera virgifera in Europe
Source: PLoS One. 2012 Nov 26;7(11):e50129. doi: 10.1371/journal.pone.0050129 (PMC3506547; doi:10.1371/journal.pone.0050129)
Supplement: Table S1 — Mean pairwise FST comparisons between Northern Italian (NW Italy, Veneto and NE Italy) and Central and South-Eastern European (CSE Europe) samples of western corn rootworm (WCR). (DOC) [file pone.0050129.s003.doc]

**Table S1:** Mean pairwise *F_ST_* comparisons between Northern Italian (NW Italy, Veneto and NE Italy) and Central and South-Eastern European (CSE Europe) samples of western corn rootworm (WCR).

| Population | NW Italy | Veneto | NE Italy | CSE Europe |
| --- | --- | --- | --- | --- |
| NW Italy | 0.01 |  |  |  |
| Veneto | 0.09 | 0.09 |  |  |
| NE Italy | 0.40 | 0.32 | - |  |
| CSE Eur | 0.26 | 0.14 | 0.14 | 0 |

Note: The population of NW Italy is represented by 3 samples (Oleggio, Storo and Fontanella), the population of Veneto is represented by 6 samples (Borso del Grappa, Conselve, Piove di Sacco, Scorze, San Donà di Piave and Summaga), the population of NE Italy is represented by 1 sample (Buttrio) and the population of CSE Europe is represented by 2 samples (Szekszard and Crepaja).
